# Cognitive Performance and Its Associations with Dental Caries: Results from the Dental, Oral, Medical Epidemiological (DOME) Records-Based Nationwide Study

**DOI:** 10.3390/biology10030178

**Published:** 2021-02-28

**Authors:** Itzhak Abramovitz, Avraham Zini, Matan Atzmoni, Ron Kedem, Dorit Zur, Noam E. Protter, Galit Almoznino

**Affiliations:** 1 Department of Endodontics, Hadassah School of Dental Medicine, Hebrew University, Jerusalem 91120, Israel; itzhakab@hadassah.org.il; 2 Department of Community Dentistry, Hadassah School of Dental Medicine, Hebrew University, Jerusalem 91120, Israel; aviz@hadassah.org.il; 3 Hebrew University, Jerusalem 91120, Israel; matanatzz@gmail.com; 4 Medical Information Department, General Surgeon Headquarter, Medical Corps, Israel Defense Forces, Tel-Hashomer 02149, Israel; ron.kedem56@gmail.com (R.K.); Dorit48@mail.idf.il (D.Z.); 5 Dental Branch and Forensic Unit, Medical Corps, Israel Defense Forces, Tel-Hashomer 02149, Israel; noamprotter@gmail.com; 6 Department of Oral Medicine, Sedation & Maxillofacial Imaging, Hadassah School of Dental Medicine, Hebrew University, Jerusalem 91120, Israel; 7 Big Biomedical Data Research Laboratory, Hadassah School of Dental Medicine, Hebrew University, Jerusalem 91120, Israel

**Keywords:** cognitive performance, intelligence quotient (IQ), caries, decayed teeth, electronic medical record, electronic dental record

## Abstract

**Simple Summary:**

Relatively few studies analyzed the association between cognitive performance and dental status. This study aimed to analyze the association between cognitive performance and dental caries. Included were data from the Dental, Oral, Medical Epidemiological (DOME) study records-based research, which integrated large socio-demographic, medical, and dental databases of a nationally representative sample of young to middle-aged military personnel (N = 131,927). The present study demonstrated that impaired cognitive performance tests were associated with worse dental status, including a higher number of decayed and missing teeth, and more teeth in need of root canal treatments and extractions. The association between cognitive performance and caries, was independent of the socio-demographic and health-related habits that were analyzed. The study concludes that better allocation of resources is recommended, focusing on populations with impaired cognitive performance in need of dental care.

**Abstract:**

Relatively few studies have analyzed the association between cognitive performance and dental status. This study aimed to analyze the association between cognitive performance and dental caries. Included were data from the dental, oral, medical epidemiological (DOME) study; cross-sectional records-based research, which integrated large socio-demographic, medical, and dental databases of a nationally representative sample of young to middle-aged military personnel (N = 131,927, mean age: 21.8 ± 5.9 years, age range: 18–50). The cognitive function of draftees is routinely measured at age 17 years using a battery of psychometric tests termed general intelligence score (GIS). The mean number of decayed teeth exhibited a gradient trend from the lowest (3.14 ± 3.58) to the highest GIS category (1.45 ± 2.19) (odds ratio (OR) lowest versus highest = 5.36 (5.06–5.68), *p* < 0.001). A similar trend was noted for the other dental parameters. The associations between GIS and decayed teeth persisted even after adjusting for socio-demographic parameters and health-related habits. The adjustments attenuated the OR but did not eliminate it (OR lowest versus highest = 3.75 (3.38–4.16)). The study demonstrates an association between cognitive performance and caries, independent of the socio-demographic and health-related habits that were analyzed. Better allocation of resources is recommended, focusing on populations with impaired cognitive performance in need of dental care.

## 1. Introduction

Intellectual performance, generally termed intelligence, combines a wide range of mental activities, such as logical reasoning, problem-solving, learning ability, and verbal skills [1]. It is a global ability that encompasses a set of numerous capabilities, behaviors, thoughts, and emotions [1]. Standardized tests were designed to assess human intelligence, resulting in a total score termed intelligence quotient (IQ). People with higher intelligence have been shown to live longer, enjoy better health, and have more favorable health behaviors [2]. Lower IQ has been associated with an increased risk of morbidity and mortality [3,4,5], while higher cognitive scores predict better general and mental health, including lower odds of having self-reported “severe tooth or gum trouble” [6].

There are relatively few studies addressing the association between cognitive function and dental status among young to middle-aged adults. The association of IQ and dental caries was previously studied mostly among children [7,8,9] and elderly populations with cognitive decline [10,11,12]. These epidemiological studies have reported the relationship between the number of teeth, mastication, periodontal disease, and dementia in elderly populations. For example, age, number of teeth, tongue pressure, and masticatory performance were significantly correlated with cognitive decline in older Japanese dental outpatients aged ≥65 years [13]. Moreover, a relationship was found between the number of teeth present and the extent of gray matter atrophy in elderly individuals with cognitive decline [14]. A systematic review concluded that older people with dementia have worse oral health, with more retained roots, and coronal and root caries [15]. Regarding the association between periodontal disease and dementia, a recent systematic review and meta-analysis concluded that periodontal disease could increase the risk of dementia, with a pooled relative risk of dementia of 1.38 (95% confidence interval: 1.01–1.90) [16]. In these elderly populations, the association was attributed to salivary gland dysfunction associated with a daily intake of medication and alcohol [17], and to poor oral hygiene mediated through a compromised capacity to perform oral health-related activities [11].

However, less studied has been the association between cognitive function and dental caries not within the context of cognitive decline in the elderly or the context of children. Moreover, the reason why these associations should be assessed in adulthood is because dental caries and periodontal disease are typical dental diseases in adulthood, and early tooth loss due to dental caries may be a factor contributing to cognitive decline. Considering this gap in the literature, the primary objective of the present study was to analyze the association between cognitive performance and dental caries among a nationally representative sample of young and middle-aged adults in Israel. An unusual opportunity exists in Israel to study the association between cognitive performance and dental status. In Israel, military service is mandatory for all eligible citizens, and therefore the military population in Israel is large and constitutes a well-grounded data source for epidemiologic research among young and middle-aged adults [18,19]. The present study used data from the dental, oral, medical epidemiological (DOME) records-based nationwide study, which captures comprehensive socio-demographic, dental, and medical databases of subjects serving in the Israel defense forces (IDF) [18,19,20,21]. The cognitive performance of draftees is routinely measured in the IDF at the age of 17 (one year before recruitment) using the battery of psychometric tests termed General Inteligeligence Score (GIS) [3,5]. The null hypothesis (H_0_) of this study was that cognitive performance test scores are not associated with a higher prevalence of dental pathologies. This study hypothesized (H_1_ hypothesis) that lower cognitive performance test scores would be associated with a higher prevalence of dental pathologies, even after adjustment for socio-demographic parameters and health-related lifestyle habits. Specific objectives of this study were to analyze the associations of GIS with the prevalence of the following dental parameters: (1) decayed teeth, (2) missing teeth, (3) the number of teeth in need of root canal treatment, and (4) the number of teeth in need of extractions. Analyses of other dental parameters beyond these are beyond the scope of this research. Decayed teeth were considered our primary outcome, and we aimed to further study their association with GIS. To that end, we used various statistical models for decayed teeth as a dependent variable adjusted for possible confounders and independent risk factors for dental caries, including: (1) socio-demographic parameters: age, sex, education, socio-economic status (SES), locality of residence, and birth country; and (2) health-related lifestyle habits: smoking, teeth brushing, cariogenic diet, and sweetened beverages.

## 2. Methods

This is part of the dental, oral, medical epidemiological (DOME) study [18,19,20,21]. The DOME is a records-based study, which consists of the socio-demographic, dental, and medical records of young to middle-aged military personnel [18,19,20,21]. Complete information on the protocol and study methods of the DOME has been published previously [18]. Data regarding GIS, dental status, sociodemographics, and health-related habits are captured in the DOME study [18]. This provides powerful means and a unique opportunity for the crossing of dental records with cognitive performance tests to explore the associations between intelligence tests and dental status, while controlling for socio-demographic variables and health-related habits.

### 2.1. Data Source

The DOME is a structured repository that captures socio-demographic, dental, and medical military databases. In the present study we used two of the DOME databases: (1) *DPR—Dental Patient Record*, which keeps all the dental information of the subjects, including dental treatment plan, actual treatments, and visits to dental care providers; (2) *IDF’s central demographic database*: which keeps the personal records of the army population, including the socio-demographic characteristics, and the GIS [18]. Data mining was executed by the Department of Medical Information of the Medical Corps anonymously [18]. The study was approved by the Medical corps Institutional board approval number 1281, 2018, with an exemption for informed consent due to anonymous data analysis.

### 2.2. Study Population

The DOME data used for this study was of military personnel, who attended the military dental clinics of the IDF, between 1 January 2015 and 1 January 2016. The study included 131,927 subjects. The mean age of the study population was 21.8 ± 5.9 years and the age range was 18–50.

### 2.3. Inclusion and Exclusion Criteria

Military personnel in mandatory and career service above 18 years of age, with the existence of data regarding the individual in the socio-demographic and the DPR databases. The absence of records (more than 5% missing data) regarding the individual in the socio-demographic database and/or the DPR was an exclusion criterion.

### 2.4. Definition of Variables

#### 2.4.1. Dependent Variables: Dental Parameters

The dental parameters were retrieved from the DPR. The standardization process of the administrative and clinical dental work up, and the quality assessment (QA) employed by the dental branch of the IDF, has been described in detail previously in the DOME methods paper [18]. Briefly, the DOME repository includes uniform codes for dental procedures that are equivalent to the nomenclature used by the American Dental Association’s (ADA) current dental terminology (CDT) [18,22]. Dental examinations were performed in an indoor setting, including bilateral bitewings for the molar and premolar areas for all dental patients, and periapical radiographs for deep caries, endodontically treated teeth, and periodontal disease [18]. The dental parameters included in the study were: the number of decayed teeth, as well as the count of missing teeth for any reason (excluding wisdom teeth) [18]. Other dental variables were included:

(1) The number of teeth in need of root canal treatments (RCT): (a) one RCT (D3310), (b) two RCT (D3320), (c) three or more RCT (D3330). The present study included the number of teeth in need of RCTs, which is the sum of these codes [18].
(2) The number of teeth in need of extractions: extraction erupted tooth or exposed root (D7140) [18].

#### 2.4.2. Independent Variables

The GIS and the socio-demographic variables were retrieved from the central socio-demographic database.

##### Assessment of the General Intelligence Score (GIS)

Before recruitment to the military at the age of 18 years, all draftees are thoroughly screened for physical and mental pathology at age 17 years, one year before mandatory military service. [3,5,23]. The screening is performed as part of the first draft to determine their eligibility for service and their suitability for various military positions [3,5,23]. The mandatory screenings contain extensive cognitive performance tests termed the general intelligence score (GIS), which is administered by personnel who have undergone a four month training course [3].

##### General Intelligence Score (GIS)

Intelligence is based on an evaluation of verbal, nonverbal, and mathematical cognitive abilities [5,18,23,24]. The validity of the GIS as a measurement of general intelligence has been demonstrated previously, with a correlation >0.8 with the Wechsler adult intelligence scale total IQ [3,25,26,27]. The GIS includes a battery of computerized psychometric tests, and is comprised of the following four subsets [3]:

- *Arithmetic-R*: assessment of mathematical reasoning, concentration, and concept manipulation.
- *Otis-R*: measurement of the understanding and execution of verbal instructions.
- *Similarities-R*: assessment of word analogies, verbal abstraction, and categorization.
- *Raven’s Progressive Matrices-R*: measurement of non-verbal abstract reasoning, and problem-solving abilities through visual–spatial shape analogies.

The sum of the test results forms a validated measure of general intelligence (IQ) scored on a 9-point scale that ranges from 10–90 [3,23]. In the present study, we analyzed the GIS as a categorical variable composed of four groups of scores: 1–3 (scores 10–30), 4–5 (scores 40–50), 6–7 (scores 60–70), and 8–9 (scores 80–90), as described previously [3,25,28,29]. The lowest GIS category (GIS 1–3) is equivalent to an IQ score range of 70–85, and draftees with these scores cannot serve in combat roles [3]. GIS 50 reflects the average score of the population, and corresponds to an IQ score of 100, whereas a value score of 90 is the highest score, and corresponds to an IQ score of 130 points and above [3].

##### Socio-Demographic Variables

The socio-demographic data included in the DOME study have been thoroughly detailed in the DOME methods paper [18]. Briefly, the following socio-demographic parameters were included: (a) age: in years, (b) sex: men/women, (c) education: high school/technicians/academics, (d) socio-economic status (SES): derived from the Israeli Ministry of the Interior records: low (1st–4th deciles)/ medium (5th–7th)/ high (8th–10th), (e) locality of residence: urban Jewish/urban non-Jewish/rural, and (f) birth: countries: Western Europe, East Europe, Former Soviet Union (FSU), Asia, East Asia, Ethiopia, Africa, North America, South America, Israel [18,21].

##### Health-Related Habits Variables

Self-reported health-related lifestyle habits (yes/no) include: current smoking, teeth brushing at least once a day; consumption of cariogenic diet (i.e., consumption of snacks and/or sweets between meals or instead of meals); and consumption of sweetened beverages (one cup or more during the day) [18,21].

### 2.5. Statistical Analysis

Data were tabulated and statistical analyses were performed using SPSS software version 25.0 (IBM, Chicago, IL, USA). Continuous variables are presented as means and standard deviations. Categorical variables are presented as frequencies and percentages.

GIS was analyzed as a categorical variable composed of the four groups of scores: 1–3, 4–5, 6–7, and 8–9. Analysis of the socio-demographic, health-related habits and dental parameters according to the GIS categories included: ANOVA (for continuous variables) and Pearson’s chi-square test or likelihood ratio test (for categorical parameters). Assessment of normal distributions of all the continuous parameters revealed a lack of normal distribution. We also used the non-parametric Kruskal–Wallis test for the continuous variables, however, there were no differences in the statistical significance between the ANOVA results and the Kruskal–Wallis test results. Considering this, and the large sample size, we presented ANOVA results.

A *p* value < 0.01 (2-tailed) was considered to indicate statistical significance due to the large sample size.

Linear regression models were used to estimate the odds ratios (ORs) and 95% confidence intervals (CIs) for dental parameters as the dependent variables among the four GIS categories. Several models were used to estimate the ORs and 95% CIs for the mean number of decayed teeth as the dependent variable across the four GIS categories, adjusted for socio-demographic parameters and health-related habits. The possible confounders and mediators of dental caries–cognitive dysfunction association are as follows:

Model 1: unadjusted; model 2: adjusted for age; model 3: adjusted for age and sex; model 4: model 3 variables and education; model 5: model 4 variables and SES; model 6: model 5 variables and locality of residence; model 7: model 6 variables and birth country; model 8: model 7 variables and smoking; model 9: model 8 variables and teeth brushing; model 10: model 9 variables and cariogenic diet; model 11: model 10 variables and sweetened beverages. The full model (model 11) was presented including multicollinearity tests. The variance inflation factors (VIFs), which are 1/Tolerance, were presented in the linear regression analysis. Although VIF >10 was considered as indicating multicollinearity, a concern may arise in weaker models when VIF >2.5, and therefore, the cutoff of VIF in the present study was 2.5.

## 3. Results

### 3.1. Socio-Demographic Characteristics and Health-Related Habits of the Study Population across the Four GIS Categories

Socio-demographic characteristics and health-related habits of the study population across the four GIS categories are presented in Table 1. The distribution of the GIS categories among the study population is as follows: 19,693 subjects (14.9%) were in GIS 1–3, 44,717 (33.9%) in GIS 4–5, 47,972 (36.4%) in GIS 6–7, and 19,545 (14.8%) in GIS 8–9 (Table 1). The following variables were associated with a higher proportion in the lower GIS groups: female sex, lower education, lower SES, urban Jewish locality, immigrant birth countries from FSU, Asia, Ethiopia, smoking, brushing teeth less than once a day, and consumption of cariogenic diet and sweetened beverages (Table 1).

### 3.2. The Dental Status of the Study Population across the Four General Intelligence score (GIS) Categories

Table 2 presents the dental status of the study population across the four GIS categories. The mean number of decayed teeth was higher in the lowest (3.14 ± 3.58) versus the highest (1.45 ± 2.19) GIS category, and the mean value of decayed teeth exhibited a gradient trend from the lowest to highest GIS categories (*p* < 0.001) (Table 2). A similar trend was noted for the other dental parameters including: missing teeth, the number of teeth in need of root canal treatment (RCTs), and the number of teeth in need of extractions (Table 2). In all these dental parameters there was a distinct and consistent gradient, whereby it was highest in the lowest GIS category and lowest in the highest.

**Figure 1:**
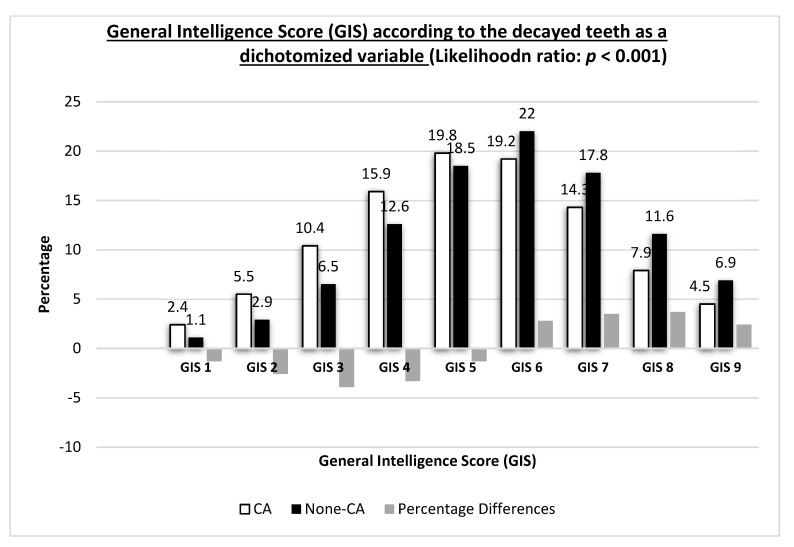
General intelligence score (GIS) according to decayed teeth as a dichotomized variable. General intelligence score (GIS), CA = presence of at least one decayed tooth; None-CA = decayed teeth were not detected on clinical examination.

We further analyzed the primary outcome of this study, i.e., decayed teeth, as a dichotomized variable: (1) CA = presence of at least one decayed tooth in the clinical examination; (2) None-CA = decayed teeth were not detected on clinical examination. The study population included 41,526 patients (35.6%) who were none-CA and 75,127 patients (64.4%) patients who were CA. As can be seen in Table 2, there was a higher proportion of participants with CA in the lowest (76.0%) versus highest (54.7%) GIS category (*p* < 0.001). To explore the cut-off point where the proportion of participants with none-CA exceeds that of CA, we further analyzed the dichotomized decayed teeth variable across all GIS categories, and the results are presented in Figure 1. GIS scores were negatively associated with the presence of decayed teeth (likelihood ratio: *p* < 0.001). For GIS scores 1–5, the differences in the prevalence between CA and none-CA were negative, i.e., higher likelihood to have at least one decayed tooth than to be without decayed teeth upon clinical examination. From the GIS score of 6–9, the differences in the scores were positive, i.e., higher likelihood to be without decayed teeth than to have at least one decayed teeth upon clinical examination (Figure 1).

Table 3 presents various linear regression models for decayed teeth (continuous variable) as the dependent variable across the four GIS categories adjusted for possible confounders and independent risk factors for dental caries. In the first unadjusted model (model 1), the OR for decayed teeth as a dependent variable was 5.36-fold higher in the lowest GIS group compared with the highest GIS group (OR = 5.36 (5.06–5.68); *p* < 0.01). ORs for decayed teeth exhibited a gradient trend from the lowest to highest GIS categories, as follows: 2.19-fold higher in the 4–5 GIS category, and 1.34-fold higher in the 6–7 GIS category compared with the highest GIS category. Further adjustments for sociodemographic parameters (models 2–7) and health-related lifestyle risk factors (models 8–11) attenuated the ORs, but did not eliminate them (see Table 3). In the final model (model number 11) which adjusted for socio-demographics, as well as for health-related lifestyle risk factors, the OR for decayed teeth was reduced compared to the unadjusted model and was 3.75-fold higher in the lowest GIS category compared with the highest GIS category (OR = 3.75 (3.38–4.16); *p* < 0.01). OR for having decayed teeth retained a gradient trend from the lowest to highest GIS categories in the last model, but with attenuated ORs as follows: 1.75-fold higher in the 4–5 GIS category, and 1.19-fold higher in the 6–7 GIS category compared with the highest GIS category (Table 3).

Table 4 presents the full linear regression model with collinearity tests of the final model (model number 11) for decayed teeth as the dependent variable. Decayed teeth retained a statistically significant positive association with (from the highest to the lowest OR): low vs. high SES (3.86 (3.36–4.44)), GIS 1–3 vs. GIS 8–9 (3.75 (3.38–4.16)), birth country East Europe vs. native Israeli (2.84 (2.28–3.55)), birth country Western Europe vs. native Israeli (1.76 (1.59–1.93)), GIS 4–5 vs. GIS 8–9 (1.75 (1.60–1.91)), brushing teeth less than once a day (1.68 (1.55–1.82)), education: technicians vs. academic (1.44 (1.25–1.69)), SES: medium vs. high (1.43 (1.36–1.52)), GIS 6–7 vs. GIS 8–9 (1.19 (1.09–1.29)), sex male vs. female (1.19 (1.12–1.26)), cariogenic diet (1.18 (1.11–1.26)), and sweetened beverages (1.009 (1.001–1.016)) (Table 4).

Decayed teeth retained a statistically significant negative association (protective parameter) with: urban non-Jewish vs. urban Jewish locality (0.78 (0.72–0.85)) and with birth country North America vs. native Israeli (0.70 (0.57–0.85)) (Table 4). As can be seen in Table 4, assessment of the collinearity between the independent variables ruled out collinearity (VIF < 2.5).

## 4. Discussion

The present study demonstrated that impaired cognitive performance tests were positively associated with worse dental status, including a higher number of decayed and missing teeth, and more teeth in need of root canal treatments and extractions. In all these dental parameters, representing caries experience in its different manifestations, there was a distinct and consistent gradient, whereby the parameter scores were highest in the lowest GIS category, and lowest in the highest. Different models were employed to study the association between GIS and our primary outcome: decayed teeth. The fact that the associations between GIS and decayed teeth persisted after adjusting for multiple confounders and independent risk factors for dental caries in this study, including sociodemographic parameters (age, sex, education, SES, locality of residence, and birth country) and health-related lifestyle habits (smoking, teeth brushing, cariogenic diet, and sweetened beverages) may support the hypothesis of an independent association between cognitive performance and the prevalence of dental caries.

The present study analyzed the association of cognitive performance tests with caries experience among a large nationally representative sample of young to middle-aged adults. The cognitive performance of draftees is routinely measured in the Israel defense forces (IDF) at the age of 17 using the battery of psychometric tests of the GIS [3,5]. Once performed, the GIS remains part of the personal records of the subject. The GIS is a valid measurement of general intelligence, and previous studies demonstrated a correlation (>0.8) with the Wechsler adult intelligence scale total IQ [3,25,26,27]. To the best of our knowledge, this is the first comprehensive study that includes a large national representative sample, where socio-demographic, dental, lifestyle habits, and cognitive tests were crossed. Previous studies have demonstrated a positive association between poor oral and dental health and low cognitive performance in middle-aged adults [17,30], and with cognitive decline in older adults [10,11,12]. Results among children are also in line with our findings, showing a strong association between IQ measured at ages 7–11 years and dental caries, with a decrease of dental caries observed in higher IQ groups [8]. Thomson et al. who measured childhood IQ also noted distinct and consistent gradients, whereby each dental caries experience measure was most severe in the lowest child IQ category and least severe in the highest [9]. Lower cognitive performance and SES were also significantly associated with higher dental treatment needs [24]. In contrast to our findings, no significant relationship was observed between IQ and dental caries among 252 children 10–15 years old [7].

The negative association between cognitive performance and dental caries could be attributed to several possible explanations. First, since cognitive performance is associated with socio-demographic factors such as education and SES, the observed inverse association between GIS and dental status could reflect these already known associations between education, SES, and IQ. Intelligence is known to play a crucial role in determining social position [31]. The associations between SES and dental needs are well established [32,33,34], and higher education has been previously shown to correlate with caries-free prevalence [35,36]. This suggests that more-intelligent people can perhaps take better care of their oral health, or are more capable of performing health-promoting actions mechanically, as they understand the concepts better. Highly-educated people may have received more knowledge about oral health and its importance, and indeed higher education levels have been shown previously to be positively associated with dental attendance [37,38]. Another possible explanation is that more intelligent people are usually of better SES and can therefore afford better dental care. This explanation is less likely in the IDF since all the participants had free access to dental care during their service regardless of their rank and position. It could reflect their experience as civilians before their recruitment, since in Israel the dental treatment of civilians is not free, and is mostly provided in fee-for-service payment models. Moreover, a study conducted in the U.S.A found that people with higher IQ and cognitive abilities visit the dentist more often regardless of SES [16]. To assess the impact of the socio-demographic parameters we analyzed the associations between GIS and dental caries in several models, and each time we adjusted the model for socio-demographic parameters in a sequential order to assess their impact on the ORs. The fact that the association between GIS and dental caries persisted even after adjustment for these socio-demographic parameters, makes this less likely to be the sole explanation, but does not exclude it.

Another explanation is that the differences in GIS could be associated with differences in lifestyle habits. Caries are associated with health-compromising lifestyle habits such as smoking, less tooth brushing, and sugar consumption [39]. Higher intelligence scores could be associated with better health-related behaviors, which could account for the observed associations. This was indeed demonstrated in the present study, as can be seen in Table 1, which shows this gradient of better health-related behaviors with higher GIS scores. In support of this, a previous study also demonstrated that better cognitive performance at ages 15–23 years predicted higher rates of dental floss use and lower rates of smoking and consumption of sugary drinks in middle age [40]. Others have also demonstrated that non-smoking and twice-daily tooth brushing and daily flossing showed the expected gradients with IQ in the opposite direction [9]. Among the elderly, the association between cognitive function and dental caries was attributed to poor oral hygiene due to compromised dentally-related function, i.e., the capacity to perform oral health-related activities (e.g., brushing teeth, scheduling dental appointments, and making treatment decisions) [11].

Alternatively, it could be the inverse associations, where lifestyle habits in adulthood, childhood, or even in utero could have influenced the intelligence scores and the subsequent risk for dental caries independently. The present study did not include past and in utero exposures to lifestyle habits, which should be included in future studies. However, as was demonstrated with socio-demographic parameters, the association between GIS and dental caries persisted even after adjustment for the health-related lifestyle risk habits. This finding may suggest the existence of other health-related lifestyle habits that were not considered, or may support an independent association between cognitive performance and caries prevalence.

Other mechanisms may be involved and results might be confounded by the effect of periodontal disease and dental caries, a typical dental disease in adulthood. Previous studies have shown that tooth loss is a risk factor for dementia, associated with the onset of dementia, particularly in the Japanese population [41,42]. Therefore, early tooth loss among young to middle-aged adults due to dental caries may be a factor. Indeed in the present study, the number of missing teeth was positively associated with the GIS score (Table 2). Reduced occlusal force and occlusion contacts due to missing teeth might be a contributor factor, considering recent studies, which suggested relationships between mild cognitive impairment (MCI) and periodontitis, gingival inflammation, oral motor skills, occlusal force, and occlusion contacts [43,44,45]. Future studies should assess these parameters longitudinally in different ages to further elucidate these possible mechanisms.

### Strength and Limitations

The major strengths of the present research are the large sample size (131,927 subjects) composed of a nationwide representative sample of young and middle-aged adults, and the use of the comprehensive database from the DOME study that captures cognitive performance tests results, socio-demographic, dental, and lifestyle habits data. Since Israel is an immigrant country multiple ethnicities were included, which may enable comparison with other populations. Uniform definitions were employed for all subjects, and all variables that were included were validated in previous publications. GIS tests are mandatory to all draftees and were measured for all subjects at the same age (17 years of age) and under the same validated protocol. Limitations include the focus of the study on a very specific population (military personnel) and possibly with a different access to health care than the general population. While multiple covariants were considered, due to the complexities of the topic other parameters were not assessed. These include parental, childhood, and in utero exposures, genetic factors, and past health-related habits. Moreover, due to the cross-sectional study design, we cannot address causality, and therefore we only discuss associations between the variables. Future longitudinal studies involving genetic and epidemiological data are recommended to reveal the origin and pathways underlying the observed findings of the present study.

## 5. Conclusions

The present study demonstrated that impaired cognitive performance tests were positively associated with a distinct and consistent gradient, whereby dental parameter scores were highest in the lowest GIS category and lowest in the highest. The associations between GIS and decayed teeth persisted even after adjusting for multiple confounders and independent risk factors for dental caries, including socio-demographic parameters and health-related lifestyle habits. The findings of the present study support the hypothesis of an association between cognitive performance and the prevalence of dental caries, independent of the socio-demographic and health-related habits that were analyzed. Based on these observations, better allocation of resources in dentistry can be established focusing on populations with impaired cognitive performance in need of dental care.

## Figures and Tables

**Table 1:** Socio-demographic characteristics and health-related habits of the study population across the four general intelligence score (GIS) categories.

Parameter		GIS Categories				Total (%) or Mean ± SD	*p* Value
		1–3	4–5	6–7	8–9		
Number (%)		19,693 (14.9)	44,717 (33.9)	47,972 (36.4)	19,545 (14.8)	131,927 (100)	
Age (years)		21.1 ± 5.1	21.5 ±6.0	22.1 ±6.1	22.6 ±6.1	21.8 ±5.9	<0.001 *
Sex	Men	15,309 (77.7)	31,774 (71.1)	35,795 (74.6)	16,107 (82.4)	98,985 (75.0)	<0.001 ^˄^
	Woman	4384 (22.3)	12,943 (28.9)	12,177 (25.4)	3438 (17.6)	32,942 (25.0)	
Education	High school	18,581 (94.6)	39,353 (88.1)	39,497 (82.4)	14,344 (73.5)	111,775 (84.8)	<0.001 ^˅^
	Technician	882 (4.5)	3415 (7.6)	2428 (5.1)	605 (3.1)	7330 (5.6)	
	Academics	182 (0.9)	1906 (4.3)	6011 (12.5)	4571 (23.4)	12,670 (9.6)	
SES	Low	1708 (8.7)	2127 (4.8)	1449 (3.1)	357 (1.9)	5641 (4.3)	<0.001 ^˅^
	Medium	12,392 (63.4)	25,450 (57.3)	22,575 (47.7)	7886 (41. 2)	68,303 (52.4)	
	High	5454 (27.9)	16,834 (37.9)	23,329 (49.3)	10,910 (57.0)	56,527 (43.3)	
Locality of residence	Urban Jewish	18,076 (92.0)	39,020 (87.5)	39,934 (83.7)	15,933 (82.2)	112,963 (86.0)	<0.001 ^˅^
	Urban non-Jewish	1569 (8.0)	5499 (12.9)	7495 (15.7)	3272 (16.9)	17,835 (13.6)	
	Rural	12 (0.1)	91 (0.2)	301 (0.6)	178 (0.9)	582 (0.4)	
Birth country	Western Europe	245 (1.2)	760 (1.7)	1075 (2.2)	592 (3.0)	2672 (2.0)	<0.001 ^˅^
	East Europe	1086 (5.5)	2755 (6.2)	2780 (5.8)	1220 (6.2)	7841 (5.9)	
	FSU	374 (1.9)	646 (1.4)	504 (1.1)	183 (0.9)	1707 (0.3)	
	Asia	52 (0.3)	98 (0.2)	62 (0.1)	24 (0.1)	236 (0.2)	
	East Asia	32 (0.2)	58 (0.1)	44 (0.1)	19 (0.1)	153 (0.1)	
	Ethiopia	1429 (7.3)	611 (1.4)	106 (0.2)	8 (0.0)	2154 (1.6)	
	Africa	40 (0.2)	104 (0.2)	125 (0.3)	50 (0.3)	319 (0.2)	
	North America	124 (0.6)	629 (1.4)	1255 (2.6)	835 (4.3)	2843 (2.2)	
	South America	133 (0.7)	335 (0.7)	323 (0.7)	165 (0.8)	956 (0.7)	
	Oceania	3 (0.0)	17(0.0)	45 (0.1)	43 (0.2)	108 (0.1)	
	Israel	16,174 (82.1)	38,698 (86.6)	41,641 (86.8)	16,396 (83.9)	112,909 (85.6)	
Smoking	No	18,507 (94.0)	42,160 (94.3)	45,641 (95.1)	18,883 (96.6)	125,191 (94.9)	<0.001 ^˄^
	Yes	1186 (6.0)	2557 (5.7)	2332 (4.9)	662 (3.4)	6736 (5.1)	
Brushing teeth once a day	No	3307 (35.3)	6316 (31.9)	5862 (29.3)	2159 (27.1)	17,644 (30.9)	<0.001 ^˄^
	Yes	6052 (64.7)	134,63 (68.1)	14,171 (70.7)	5810 (72.9)	39,496 (69.1)	
Cariogenic diet	No	3083 (43.4)	7326 (47.6)	8046 (50.8)	3448 (53.7)	21,903 (48.9)	<0.001 ^˄^
	Yes	4024 (56.6)	8049 (52.4)	7808 (49.2)	2978 (46.3)	22,859 (51.1)	
Sweetened beverages	No	4593 (49.1)	10,946 (55.3)	12,080 (60.3)	5150 (64.6)	32,769 (57.3)	<0.001 ^˄^
	Yes	4766 (50.9)	8833 (44.7)	7953 (39.7)	2819 (35.4)	24,371 (42.7)	

* ANOVA, ^^^ Pearson’s chi-square, ^˅^ Likelihood ratio, FSU: Former Soviet Union.

**Table 2:** Dental status of the study population across the four general intelligence score (GIS) categories.

Parameter		GIS Categories (Mean ± SD or (%))				Total Mean ± SD or (%)	*p* Value *
		1–3	4–5	6–7	8–9		
Decayed teeth	Mean ± SD	3.14 ± 3.58	2.25 ± 2.87	1.75 ± 2.43	1.45 ± 2.19	2.09 ± 2.81	<0.001 *
	OR & 95% CI	5.36 (5.06–5.68)	2.19 (2.08–2.30)	1.34 (1.27–1.41)	1		
Missing teeth	Mean ± SD	0.63 ± 1.24	0.58 ± 1.25	0.57 ± 1.38	0.51 ± 1.17	0.58 ± 1.29	<0.001 *
	OR & 95% CI	1.65 (1.61–1.70)	1.07 (1.04–1.11)	1.06 (1.03–1.10)	1		
Number of teeth in need of root canal treatment	Mean ± SD	0.18 ± 0.60	0.09 ± 0.37	0.05 ± 0.28	0.04 ± 0.25	0.08 ± 0.37	<0.001 *
	OR & 95% CI	1.15 (1.14–1.16)	1.05 (1.04–1.05)	1.01 (1.00–1.01)	1		
Number of teeth in need of extractions	Mean ± SD	0.22 ± 0.69	0.14 ± 0.52	0.11 ± 0.45	0.11 ± 0.45	0.14 ± 0.52	<0.001 *
	OR & 95% CI	1.12 (1.11–1.13)	1.03 (1.02–1.04)	1.00 (0.99–1.01)	1		
Decayed teeth as a dichotomized variable	CA	13,811 (76.0)	26,844 (67.5)	25,188 (60.4)	9284 (54.7)	75,127 (64.4)	<0.001 **
	None-CA	4361 (24.0)	12,936 (32.5)	16,542 (39.6)	7687 (45.3)	41,526 (35.6)	

* ANOVA, ** Pearson’s chi-square, general intelligence score (GIS), CA = presence of at least one decayed tooth; None-CA = decayed teeth were not detected on clinical examination.

**Table 3:** Odds ratios (ORs) for decayed teeth as the dependent variable across four general intelligence score (GIS) categories for various models.

Variable	GIS Categories			
	1–3	4–5	6–7	8–9
**Model 1-unadjusted decayed teeth vs. GIS categories**				
OR and 95% CI	5.36 (5.06–5.68)	2.19 (2.08–2.30)	1.34 (1.27–1.41)	1
*p* value	<0.001	<0.001	<0.001	
**Model 2-Model 1 adjusted for age**				
OR and 95% CI	5.27 (4.97–5.58)	2.22 (2.11–2.33)	1.34 (1.29–1.42)	1
*p* value	<0.001	<0.001	<0.001	
**Model 3-adjusted for age and sex**				
OR and 95% CI	5.23 (4.93–5.54)	2.15 (2.05–2.26)	1.33 (1.26–1.39)	1
*p* value	<0.001	<0.001	<0.001	
**Model 4-adjusted for age, sex and education**				
OR and 95% CI	5.11 (4.81–5.42)	2.18 (2.07–2.29)	1.33 (1.27–1.41)	1
*p* value	<0.001	<0.001	<0.001	
**Model 5-adjusted for age, sex and education and SES**				
OR and 95% CI	4.25 (4.00–4.52)	1.96 (1.86–2.07)	1.28 (1.21–1.34)	1
*p* value	<0.001	<0.001	<0.001	
**Model 6-adjusted for age, sex and education, SES, and locality**				
OR and 95% CI	4.12 (3.88–4.38)	1.93 (1.83–2.03)	1.27 (1.21–1.34)	1
*p* value	<0.001	<0.001	<0.001	
**Model 7-adjusted for age, sex and education, SES, locality, and birth country**				
OR and 95% CI	4.16 (3.91–4.43)	1.93 (1.83–2.03)	1.27 (1.21–1.34)	1
*p* value	<0.001	<0.001	<0.001	
**Model 8-adjusted for age, sex and education, SES, locality, birth country, and smoking**				
OR and 95% CI	4.13 (3.89–4.39)	1.92 (1.82–2.02)	1.27 (1.21–1.33)	1
*p* value	<0.001	<0.001	<0.001	
**Model 9-adjusted for age, sex and education, SES, locality, birth country, smoking, and teeth brushing**				
OR and 95% CI	4.14 (3.73–4.58)	1.86 (1.70–2.03)	1.22 (1.12–1.33)	1
*p* value	<0.001	<0.001	<0.001	
**Model 10-adjusted for age, sex and education, SES and locality, birth country, smoking, teeth brushing, and cariogenic diet**				
OR and 95% CI	4.02 (3.63–4.46)	1.82 (1.67–1.99)	1.21 (1.11–1.32)	1
*p* value	<0.001	<0.001	<0.001	
**Model 11-adjusted for age, sex and education, SES and locality, birth country, smoking, teeth brushing, cariogenic diet, and sweetened beverages**				
OR and 95% CI	3.75 (3.38–4.16)	1.75 (1.60–1.91)	1.19 (1.09–1.29)	1
*p* value	<0.001	<0.001	<0.001	

**Table 4:** Model number 11: linear regression analysis with collinearity tests for decayed teeth as the dependent variable.

Parameter	B	Std. Error	*p* Value	Exp(B) and 95% Wald Confidence Interval for	Collinearity Statistics	
					Tolerance	VIF
(Intercept)	1.88	0.16	<0.001	6.59 (4.79–9.06)		
GIS 1–3 vs. GIS 8–9	1.32	0.05	<0.001	3.75 (3.38–4.16)	0.74	1.34
GIS 4–5 vs. GIS 8–9	0.56	0.04	<0.001	1.75 (1.60–1.91)	0.74	1.34
GIS 6–7 vs. GIS 8–9	0.17	0.04	<0.001	1.19 (1.09–1.29)	0.80	1.24
Age	−0.009	0.003	0.021	0.99 (0.98–0.99)	0.38	2.39
Sex: Male vs. female	0.17	0.03	<0.001	1.19 (1.12–1.26)	0.94	1.06
Education: high school vs. academic	0.15	0.06	0.460	1.05 (0.92–1.20)	0.69	1.43
Education: technicians vs. academic	0.37	0.07	<0.001	1.44 (1.25–1.69)	0.48	2.05
SES: low vs. high	1.35	0.07	<0.001	3.86 (3.36–4.44)	0.95	1.05
SES: medium vs. high	0.36	0.02	<0.001	1.43 (1.36–1.52)	0.92	1.07
Locality: Urban non-Jewish vs. urban Jewish	−0.24	0.04	<0.001	0.78 (0.72–0.85)	0.97	1.02
Locality: Rural vs. urban Jewish	0.53	0.32	0.106	1.70 (0.89–3.23)	0.98	1.01
Birth country Western Europe vs. native Israeli	0.56	0.05	<0.001	1.76 (1.59–1.93)	0.98	1.01
Birth country East Europe vs. native Israeli	1.04	0.11	<0.001	2.84 (2.28–3.55)	0.98	1.01
Birth country Asia vs. native Israeli	0.21	0.22	0.350	1.23 (0.79–1.92)	0.99	1.002
Birth country Ethiopia vs. native Israeli	−0.22	0.11	0.040	0.80 (0.64–0.98)	0.96	1.04
Birth country Africa vs. native Israeli	−0.19	0.28	0.946	0.98 (0.56–1.71)	0.99	1.001
Birth country North America vs. native Israeli	−0.35	0.10	<0.001	0.70 (0.57–0.85)	0.99	1.01
Birth country South America vs. native Israeli	−0.33	0.16	0.837	0.96 (0.70–1.32)	0.99	1.002
Smoking	0.026	0.07	0.704	1.02 (0.89–1.17)	0.79	1.25
Brushing teeth less than once a day	0.52	0.04	<0.001	1.68 (1.55–1.82)	0.98	1.01
Cariogenic diet	0.17	0.03	<0.001	1.18 (1.11–1.26)	0.72	1.37
Sweetened beverages	0.45	0.03	<0.001	1.009 (1.001–1.016)	0.71	1.40

## Data Availability

Data sharing not applicable.
